# A Rare Presentation of Appendiceal Carcinoma

**DOI:** 10.7759/cureus.16370

**Published:** 2021-07-13

**Authors:** Kevin Walsh, Niranjan Ojha, Amitpal Nat, Lorenzo Gitto, Vesna Untanu

**Affiliations:** 1 Internal Medicine, State University of New York Upstate Medical University, Syracuse, USA; 2 Pathology, State University of New York Upstate Medical University, Syracuse, USA

**Keywords:** appendiceal cancer, anemia, hemicolectomy, appendicitis, iron deficiency

## Abstract

Appendiceal cancer is a rare malignancy. Our patient presented initially to her primary care physician due to symptoms of lightheadedness and dizziness and was found to have severe anemia requiring hospital admission. She underwent a colonoscopy and was found to have mucosal ulceration in the appendiceal orifice. She underwent a biopsy of the ulceration, which was remarkable for moderately differentiated adenocarcinoma. The patient then underwent a right hemicolectomy. The usual presentation for appendiceal carcinoma is acute appendicitis; however, our patient presented with the microcytic anemia.

## Introduction

Appendiceal cancer is rare with an incidence of 0.12 cases per 100,000 people per year [1]. Furthermore, the survival rate for people diagnosed with appendiceal cancer is low due to late detection, as many individuals remain asymptomatic for years [1]. Patients with appendiceal cancer most commonly present with signs and symptoms of acute appendicitis [2]. In our case, we present an elderly female who had a rare presentation of appendiceal cancer where she presented with acute blood loss anemia. Adenocarcinoma of the colon commonly presents with iron deficiency anemia secondary to blood loss, and that is how our patient presented to the hospital. Even though she presented with a similar presentation as adenocarcinoma of the colon, the fact remains that appendiceal cancer is still a rare malignancy.

## Case presentation

A 77-year-old female with a past medical history of hypothyroidism and a past surgical history of cholecystectomy presented to the hospital as a transfer from a local community hospital for further evaluation of acute severe anemia. About a week prior to the presentation, the patient’s niece mentioned that the patient was intermittently feeling lightheaded and dizzy upon standing. Two days before admission, the patient went to her primary doctor for an annual physical examination and had laboratory tests done. After the tests resulted, it was seen that the patient had severe anemia, and she was instructed by her primary care physician to go to the local hospital for further evaluation. At the local hospital, a complete blood count revealed a hemoglobin/hematocrit of 4.6/19.4 and a mean corpuscular volume (MCV) of 56.9. Additionally, a fecal occult blood test was completed and was positive. She received two units of packed red blood cells at the transferring hospital and was subsequently transferred.

On presentation after the transfer, the patient was hemodynamically stable. Vital signs revealed: 36.6 F, heart rate: 80, respiratory rate: 18, blood pressure: 120/60, O_2_ saturation: 100%. On physical exam, the patient appeared pale and conjunctival pallor was noted. Labs were remarkable for white blood cell count 7.9, hemoglobin/hematocrit (H/H) 7.9/26.0, platelets 417, MCV 61.6, red cell distribution width 33.6, prothrombin/INR 14.5/1.12. A complete blood count from 2018 revealed H/H 11.0/35.0 and MCV 90.9. Iron studies post-transfusion revealed iron 150, total iron-binding capacity 433, ferritin 26, % Fe saturation 35.0. The complete metabolic panel was only remarkable for bicarbonate of 21.

In further history gathering, the patient mentioned that her stool had been darker than usual over the past two weeks but denied any change in bowel habits, abdominal pain, or bleeding episodes. She did endorse fatigue and generalized weakness over the past month as well. The patient denied any history of tobacco use, alcohol use, and in terms of health maintenance, she had never undergone a colonoscopy.

**Figure 1:**
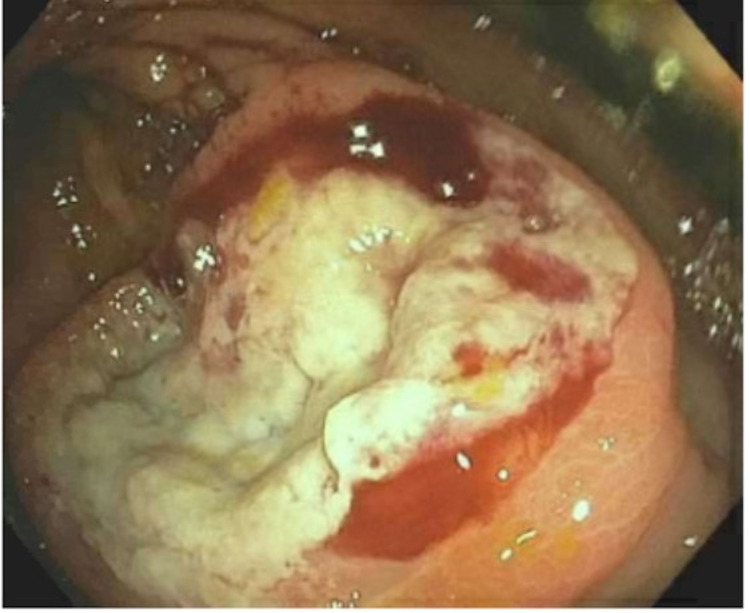
Mucosal ulceration in appendiceal orifice (picture taken from colonoscopy).

She was made NPO (nothing through mouth), IV fluids were started, as well as IV Protonix 40 mg BID. The patient was admitted, and gastroenterology was consulted, recommending esophagogastroduodenoscopy (EGD) and colonoscopy. EGD was completed and revealed gastric mucosal atrophy, a nodule in the esophagus, and normal duodenum. Biopsies of the duodenum, stomach, and esophagus were taken and were only remarkable for mild chronic gastritis involving the stomach. A colonoscopy was completed and revealed non-bleeding internal hemorrhoids and diverticulosis at the recto-sigmoid colon. Mucosal ulceration found at the appendiceal orifice was biopsied (Figure 1).

A CT abdomen/pelvis was completed to further evaluate the appendiceal orifice ulceration and was remarkable for a 29 mm area of low density along the inferior aspect of the right liver lobe and a larger lesion within the right liver lobe measuring 21 mm. Ultrasound abdomen was then obtained to characterize further the hepatic lesions, which revealed a hyperechoic lesion in the right hepatic lobe measuring 2.4 x 1.8 x 2.2 cm consistent with a hemangioma. Ultrasound abdomen also revealed a hypoechoic lesion in the right hepatic lobe dome measuring 3.5 x 2.1 x 3.3 cm, which might represent a complex cyst. During the admission, an alpha-fetoprotein, carcinoembryonic antigen (CEA), and CA 19-9 were obtained and remarkable for CEA 7.9 ng/mL (normal range 0-2.5 ng/mL). To further characterize the hepatic lesions, an MRI with the liver mass protocol was obtained and revealed T2 hyperintense lesions in the right hepatic lobe, 1.1 cm T2 hyperintense lesion in the hepatic dome, 1.4 cm and 1 cm T2 hyperintense lesions and 7 and 6 mm T2 hyperintense foci in the left hepatic lobe.

**Figure 2:**
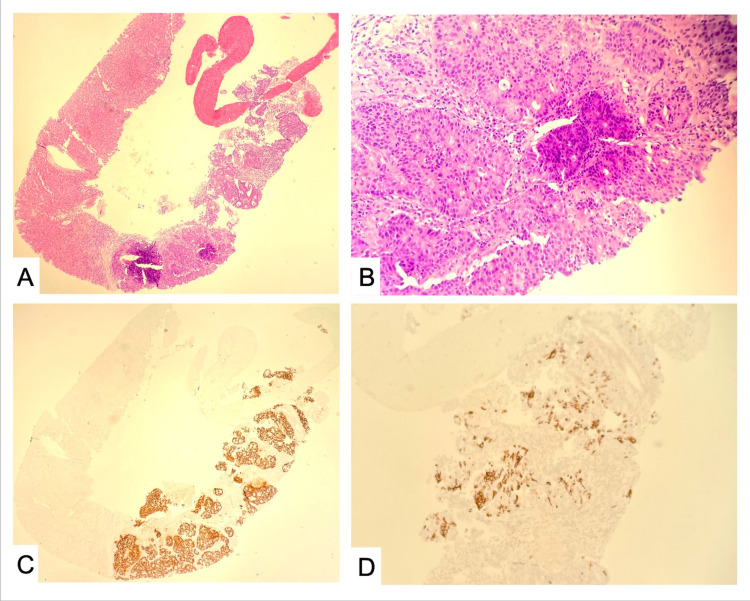
Microscopic examination showed metastatic adenocarcinoma (A, B – H&E, 40x and 200x). The tumor cells stained positive for CDX2 (C) and CK20 (D, partial) and negative for CK7.

The biopsy taken from the colonoscopy resulted on day 5 of the admission and was remarkable for moderately differentiated adenocarcinoma. Hematology/oncology was consulted and recommended CT head, CT thorax, and bone scan to evaluate for metastasis, the results of which were negative. Hematology/oncology also recommended completing Interventional radiology guided biopsy of the liver lesions, which revealed metastatic adenocarcinoma compatible with appendiceal primary. The tumor cells were positive for CK20 (partial) and CDX2 and negative for CK7 (Figure 2). Surgical oncology was consulted and recommended surgical excision of the lesion to prevent further gastrointestinal losses as the patient presented with severe blood loss anemia. The patient ultimately underwent a right hemicolectomy. The patient did well after surgery and was subsequently discharged with hematology/oncology follow-up outpatient. At the patient’s outpatient hematology/oncology appointment, treatment options were discussed, and the patient refused any treatment and opted for hospice care.

## Discussion

Our patient had a rare presentation of appendiceal cancer, where she presented with acute blood loss anemia. Most undiagnosed appendiceal cancer patients usually present with acute appendicitis signs and symptoms, as mentioned previously [2]. Patients also usually present with liver or peritoneal metastasis [3]. This type of malignancy has a poor survival rate due to late diagnosis [1]. The average age at diagnosis for appendiceal cancer is 50-55 years, and the malignancy affects men and women equally [4]. The cause or risk factors for appendiceal cancer are unknown, and it does not run in families [4]. In our case, the patient was diagnosed with metastatic appendiceal cancer due to a late diagnosis as she did not exhibit signs or symptoms until late. Treatment usually involves surgery, including either hemicolectomy, appendectomy, or cytoreductive surgery, and chemotherapy [5].

According to the National Organizations for Rare Diseases (2018), the most common appendiceal cancer subtype is neuroendocrine or carcinoid tumors of the appendix that are derived from enterochromaffin cells [4]. These tumor types are usually found during surgery for acute appendicitis or other surgical procedures [6]. The other subtype of appendiceal cancer includes the epithelial type, which arises from gland forming cells in the appendix and is further classified into subtypes depending on the microscopic examination of the cells [4]. The categories of epithelial carcinoma include Goblet Cell, Low-Grade Mucinous Neoplasm of the Appendix, High-Grade Mucinous Neoplasm of the Appendix, and Adenocarcinoma with appendiceal adenocarcinoma further classified into well-differentiated, moderately differentiated, poorly differentiated, or signet ring cell adenocarcinoma [4]. In our patient, the biopsy report revealed that she had moderately differentiated adenocarcinoma of the appendix.

In a study completed by Marmor et al., the group identified all appendiceal cancers in the SEER registry from 2000 to 2009 [1]. They divided the tumor types into two histological groups: carcinoid and adenocarcinoma. The group further divided adenocarcinoma into adenocarcinoma not otherwise specified, mucinous, and signet ring cell. They excluded cases diagnosed before age 18 or after age 80, cancers diagnosed by autopsy, reports from nursing homes, and those without microscopic examination. The group identified 4,765 cases of appendiceal cancer. The most common type identified was mucinous adenocarcinoma at 38%, followed by carcinoid at 28%, adenocarcinoma-not otherwise specified at 27%, and finally, signet ring cell adenocarcinoma at 7%. In the study period 2000-2009, the group found that the incidence rate per 100,000 population increased from 0.63 in 2000 to 0.97 in 2009. The study group attributed the increased incidence to an increase in colonoscopy and CT scanning; however, they reasoned that the increase in scanning and colonoscopy could not account for that high increase. Further investigation was warranted. Furthermore, the study found that 74% of the appendiceal cancers were diagnosed with either regional (39%) or distant metastasis (34%). The group identified that mucinous (51%) and signet ring cells (60%) were more likely to have a distant disease at diagnosis than carcinoid (15%) and adenocarcinoma-not otherwise specified (25%). Our patient was diagnosed with distant metastasis, and the biopsy revealed moderately differentiated adenocarcinoma.

Our patient underwent a right hemicolectomy to treat appendiceal cancer and to control her gastrointestinal bleeding. She then ultimately decided on hospice care. Our patient had metastatic appendiceal cancer and typical treatment of this type of metastatic cancer includes removal of the tumor by surgery followed by chemotherapy [7]. Our patient opted for hospice care after surgery therefore she did not receive any chemotherapy. Radiation therapy is rarely used in appendiceal cancer and when it is used it is mainly when cancer spreads to the bone to relieve symptoms [7].

A study completed by Ito et al. examined long-term outcomes after surgical therapy for appendiceal cancer [8]. The group examined retrospective outcomes after surgical therapy at a tertiary medical center from 1981 to 2001. A total of 36 patients were identified and treated. The patient population included 22 individuals who were women, and the mean age was 52 years [8]. Eighty-eight percent of the patients presented with signs and symptoms of acute appendicitis. Eighteen patients underwent curative resection, including seven primary right hemicolectomies, 10 appendectomies plus subsequent right hemicolectomy, and one appendectomy [8]. The mean length at follow-up was 55 months, and the five-year survival rate was 46%. The study group found that factors associated with improved five-year survival included T stage (75 vs. 47% for T1 and 2 vs. T3 and 4, respectively, p<0.05). Tumor grade also impacted five-year survival by 100 vs. 46% for well-differentiated tumors vs. moderately or poorly differentiated tumors, respectively (p<0.05) [8]. Ultimately, patients diagnosed earlier and without distant spread have better outcomes than those diagnosed late.

## Conclusions

Appendiceal cancer remains a rare malignancy and is often diagnosed late with regional or distant spread leading to a poor survival rate. Our patient had a unique presentation of a very rare malignancy. It has not been reported previously that a patient presented with acute blood loss anemia and was found to have an appendiceal malignancy. Our patient underwent a right hemicolectomy and then opted for hospice care. At the time of diagnosis, our patient already had a distant spread. Earlier diagnosis is vital to improving patient survival and outcomes.

## References

[1] Marmor S, Portschy PR, Tuttle TM, Virnig BA (2015). The rise in appendiceal cancer incidence: 2000-2009. J Gastrointest Surg.

[2] Todd RD, Sarosi GA, Nwariaku F, Anthony T (2004). Incidence and predictors of appendiceal tumors in elderly males presenting with signs and symptoms of acute appendicitis. Am J Surg.

[3] Bhat AP, Schuchardt PA, Bhat R, Davis RM, Singh S (2019). Metastatic appendiceal cancer treated with Yttrium 90 radioembolization and systemic chemotherapy: a case report. World J Radiol.

[4] (2020). Appendiceal cancer and tumors. https://rarediseases.org/rare-diseases/appendiceal-cancer-tumors/.

[5] (n.d.). Treatment for appendiceal cancer. https://www.mskcc.org/cancer-care/types/appendiceal/treatment.

[6] Roggo A, Wood WC, Ottinger LW (1993). Carcinoid tumors of the appendix. Ann Surg.

[7] (2019). Appendix cancer: types of treatment. https://www.cancer.net/cancer-types/appendix-cancer/types-treatment.

[8] Ito H, Osteen RT, Bleday R, Zinner MJ, Ashley SW, Whang EE (2004). Appendiceal adenocarcinoma: long-term outcomes after surgical therapy. Dis Colon Rectum.

